# Increasing the length and hydrophobicity of the C‐terminal sequence of transthyretin strengthens its binding affinity to retinol binding protein

**DOI:** 10.1002/2211-5463.12329

**Published:** 2017-11-16

**Authors:** Rattawan Poodproh, Supavadee Kaewmeechai, Ladda Leelawatwattana, Porntip Prapunpoj

**Affiliations:** ^1^ Department of Biochemistry Faculty of Science Prince of Songkla University Hat Yai Thailand

**Keywords:** binding affinity, retinol binding protein, transthyretin

## Abstract

Transthyretin (TTR) is a transporter for thyroid hormone (TH) and retinol, the latter via binding with retinol binding protein (RBP). Both the N‐terminal and C‐terminal regions of the TTR subunit are located in close proximity to the central binding channel for ligands. During the evolution of vertebrates, these regions changed in length and hydropathy. The changes in the N‐terminal sequence were demonstrated to affect the binding affinities for THs and RBP. Here, the effects of changes in the C‐terminal sequence were determined. Three chimeric TTRs, namely pigC/huTTR (human TTR with the C‐terminal sequence changed to that of *Sus scrofa* TTR), xenoN/pigC/huTTR (human TTR with the N‐terminal and C‐terminal sequences changed to those of *Xenopus laevis* and *S. scrofa*, respectively), and pigC/crocTTR (*Crocodylus porosus* TTR with the C‐terminal sequence changed to that of *S. scrofa* TTR), were constructed and their binding affinities for human RBP were determined at low TTR/RBP molar ratio using chemiluminescence immunoblotting. The binding dissociation constant (*K*_d_) values of pigC/huTTR, xenoN/pigC/huTTR and pigC/crocTTR were 3.20 ± 0.35, 1.53 ± 0.38 and 0.31 ± 0.04 μm, respectively, and the *K*_d_ values of human and *C. porosus* TTR were 4.92 ± 0.68 and 1.42 ± 0.45 μm, respectively. These results demonstrate chimeric TTRs bound RBP with a higher strength than wild‐type TTRs, and the changes in the C‐terminal sequence of TTR had a positive effect on its binding affinity for RBP. In addition, changes to the N‐terminal and C‐terminal sequences showed comparable effects on the binding affinity.

**Abbreviations**
ECL: enhanced chemiluminescence
*K*_d_: dissociation constant
RBP: retinol binding protein
TH: thyroid hormone
TTR: transthyretin

Transthyretin (TTR) is a homotetrameric protein present in serum and cerebrospinal fluid of most vertebrates. In humans, TTR is mainly synthesized by the liver and the choroid plexus of the brain. Each of its subunits comprises 127 amino acid residues 1, 2. The major function of TTR is as a transporter for thyroid hormones (THs) 3, 4 and retinol, the latter via binding to retinol binding protein (RBP) 5. In the blood circulation, the majority of the retinol forms a complex with RBP and then binds to TTR to form a retinol–RBP–TTR complex (for reviews, see 6, 7). The binding with TTR protects RBP from early clearance by glomerular filtration in the kidney and prevents RBP from binding to surface signaling receptor STRA6, which leads to the inhibition of STRA6‐inducible cellular uptake of an excessive holo‐retinol 8. Based on the crystallography of the TTR–RBP complex, the amino acids involved in the binding interaction are located in the C‐terminal regions of both TTR and RBP polypeptides 9, 10.

During the evolution of vertebrates, the amino acid sequence in the central domain of TTR monomer was highly conserved. The predominant changes were in the N‐terminal region, with smaller changes in the C‐terminal region of the TTR subunit 11, 12, 13, 14, 15, 16, 17. The N‐terminal sequences of birds, reptiles, amphibians and fish are longer or relatively more hydrophobic than those of mammalian TTRs. The C‐terminal sequences of non‐mammalian TTRs are more hydrophobic than that of human TTR; in addition, the TTRs of some vertebrates, including *Sus scrofa*, are a few amino acids longer compared with human TTR. By domain substitution experiments among TTRs, we previously demonstrated that length and hydrophobicity of the N‐terminal sequence influenced the binding affinities for THs 18 and RBP 19. In addition, the changes of binding to THs agreed well with the shift of TH binding preference from T3 to T4 during the evolution of vertebrate TTRs 20. Based on the fact that both N‐terminal and C‐terminal regions of TTR are located in close proximity at the entrance to the central binding channel for THs and other ligands, we proposed that the evolutionary change of the C‐terminal sequence influenced the function of TTR as a cotransporter for retinol.

According to the alignment of identified mature TTR polypeptides, the N‐terminal sequence of *Crocodylus porosus* TTR is in length and hydrophobicity between human and *Xenopus laevis* TTRs. In addition, the C‐terminal sequence of *S. scrofa* TTR is longer and more hydrophobic than those of human and *C. porosus* TTRs 11, 16. To demonstrate the effect of the evolutionary changes in the C‐terminal sequence of TTR on its binding interaction with RBP, and to compare the effect with that of the changes in the N‐terminal sequence, we produced two chimeric TTRs in which C‐terminal sequences were changed to be longer and more hydrophobic as observed in *S. scrofa* TTR. These were pigC/huTTR and pigC/crocTTR. The chimeric TTR xenoN/pigC/huTTR in which N‐terminal and C‐terminal sequences were simultaneously changed to those of *X. laevis* and *S. scrofa* TTRs, respectively, was also produced by using the heterologous gene expression system of *Pichia pastoris*. The binding affinities to human RBP of these chimeric TTRs were determined and compared with their wild‐type TTRs. Based on the dissociation constant (*K*_d_) values of the binding, the effects of the N‐terminal and the C‐terminal sequences on the binding affinity of TTR to RBP were comparable.

## Materials and methods

### Synthesis and purification of TTRs

Recombinant *C. porosus* TTR and chimeric TTRs, namely xenoN/pigC/huTTR (which consisted of amino acid residues 1–9 of *X. laevis* TTR, residues 10–120 of human TTR and residues 121–130 of *S. scrofa* TTR), pigC/huTTR (which consisted of amino acid residues 1–120 of human TTR and residues 121–130 of *S. scrofa* TTR), and pigC/crocTTR (which consisted of amino acid residues 1–114 of *C. porosus* TTR and residues 115–130 of *S. scrofa* TTR), were produced from recombinant *P. pastoris* G115 clones cultured in buffered glycerol complex medium and buffered methanol complex medium as previously described 14. The gene expression of TTR was induced with 0.5% methanol for 3 days. Then, the recombinant TTR, which was secreted into the culture medium, was purified by preparative native PAGE (12% resolving and 4% stacking gels) using Bio‐Rad (Hercules, CA, USA) Prep Cell (Model 491). Purified TTR was made sterile by filtering through a 0.2 μm membrane, aliquoted and stored at −20 °C until use.

### Purification of human TTR from plasma

Human TTR was purified from plasma by affinity chromatography on a Cibacron blue 3GA column (Sigma‐Aldrich, St Louis, MO, USA) and followed by preparative native PAGE, as previously described 19. The protein concentration was determined by Lowry's method 21 prior to filter sterilization through a 0.2 μm membrane and storage at −20 °C.

### Preparation of polyclonal antibody against *C. porosus* TTR

The specific polyclonal antibody for *C. porosus* TTR was produced in a male rabbit (New Zealand White; 3 months old) as previously described 19. After the second booster dose, blood (20 mL) was collected and the specific titer was determined by Ouchterlony double immunodiffusion 22. The antibody was partially purified by precipitation with 50% ammonium sulfate, aliquoted and stored at −20 °C until use.

### The analysis of TTR by electrophoresis

The mobility under native conditions and the subunit mass of TTR were determined by native PAGE (10% resolving and 4% stacking gels) and SDS/PAGE (12% resolving and 4% stacking gels), respectively. The protein bands were visualized after staining with Coomassie Brilliant Blue R‐250.

### The binding interaction between TTR and human RBP

The binding interaction between TTR and human RBP was examined as previously described 19, at a lower molar ratio of TTR/RBP than in the previous report. Purified TTR (0.5 μm) was incubated with human RBP (0–4 μm) at 4 °C for 2 h. Then, the separation of proteins in the reaction mixture was performed in duplicate by native PAGE (10% polyacrylamide resolving gel). After the separation, one gel was stained with Coomassie Brilliant Blue to detect the protein bands, and proteins in the other gel were electrotransferred onto a Hybond‐enhanced chemiluminescence (ECL) membrane (Amersham Pharmacia Biotech, Piscataway, NJ, USA), at 25 V for 30 min. Non‐specific binding sites on the membrane was blocked with 5% non‐fat dry milk. The free and RBP‐bound forms of TTR were determined by western analysis and followed by ECL detection, using sheep anti‐human TTR (dilution 1 : 2500) or rabbit anti‐*C. porosus* TTR (dilution 1 : 500) as primary antibody, and horseradish peroxidase‐conjugate anti‐sheep IgG or anti‐rabbit IgG (dilution 1 : 2500) as secondary antibody.

The *K*_d_ of the binding interaction between TTR and RBP was analyzed by Scatchard analysis 23. Bound TTR was calculated from the fluorescence intensity of the protein band based on the assumption that TTR and RBP formed a complex at 1 : 2 molar ratio. The corrections for the efficiency of protein transfer and non‐specific binding were performed prior to Scatchard analysis. Least squares linear regression lines were calculated using Microsoft Excel, and *K*_d_ values were derived from the slopes. The differences of the *K*_d_ values were determined by one‐way ANOVA.

## Results

### Physicochemical properties of chimeric TTRs

**Figure 1:**
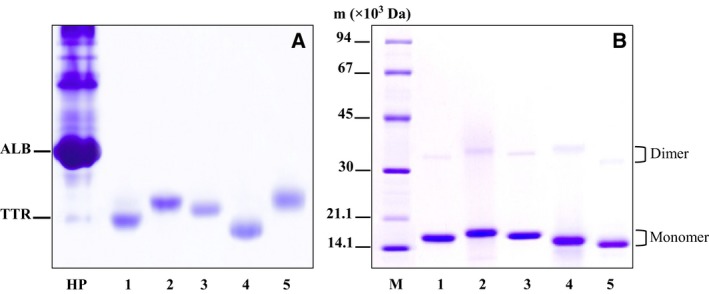
The analysis of purified TTRs by native PAGE (A) and SDS/PAGE (B). Aliquots of purified human TTR (1), xenoN/pigC/huTTR (2), pigC/huTTR (3), *Crocodylus porosus* TTR (4) and pigC/crocTTR (5) were loaded onto the gels. For SDS/PAGE, the protein sample was boiled in the presence of SDS and β‐mercaptoethanol at final concentrations of 2% and 2.5%, respectively, for 30 min. After analysis, gels were stained with Coomassie Brilliant Blue R‐250. HP, human plasma with an excess amount was included to locate the positions of albumin (ALB) and TTR; M, low molecular mass protein markers. Dimer and monomer positions of TTR are indicated.

By native PAGE, it was shown that all of the studied TTRs migrated faster than albumin (ALB) in human plasma (HP; Fig. 1A). Under the denaturing condition of SDS/PAGE, only a major and a minor protein band were observed in each studied TTR, and these two bands migrated with a relative mobility corresponding to the monomer and dimer of TTR, respectively (Fig. 1B). The molecular masses of the subunit of the studied TTRs were in range of 14.5–18.1 kDa, and the masses of the dimeric forms were ~ 31 kDa. According to high performance liquid chromatography analysis, the molecular mass of the tetrameric TTR was ~ 4 times the subunit mass (data not shown), which is in the range of that reported for human and *C. porosus* wild‐type TTRs 14.

### Analysis of the binding between TTR and human RBP

**Figure 2:**
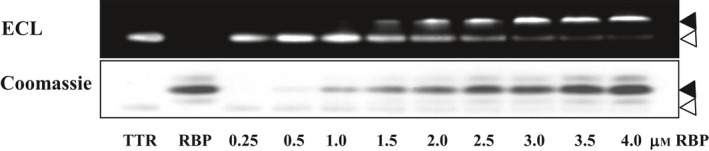
Analysis of binding between TTR and human RBP by native PAGE followed by western analysis. Purified TTR (0.5 μm) was incubated with human RBP at various concentrations. Then, free and bound TTRs were separated by native PAGE in duplicate. One of the gels was stained with Coomassie Brilliant Blue R‐250, and another gel was subjected to western blot analysis using antibody specific to human TTR or *Crocodylus porosus* TTR, and followed by ECL detection. Purified TTR (0.5 μm; TTR) and human RBP (4 μm; RBP) were included as controls. Free and bound TTRs are indicated by closed and opened arrowheads, respectively.

**Figure 3:**
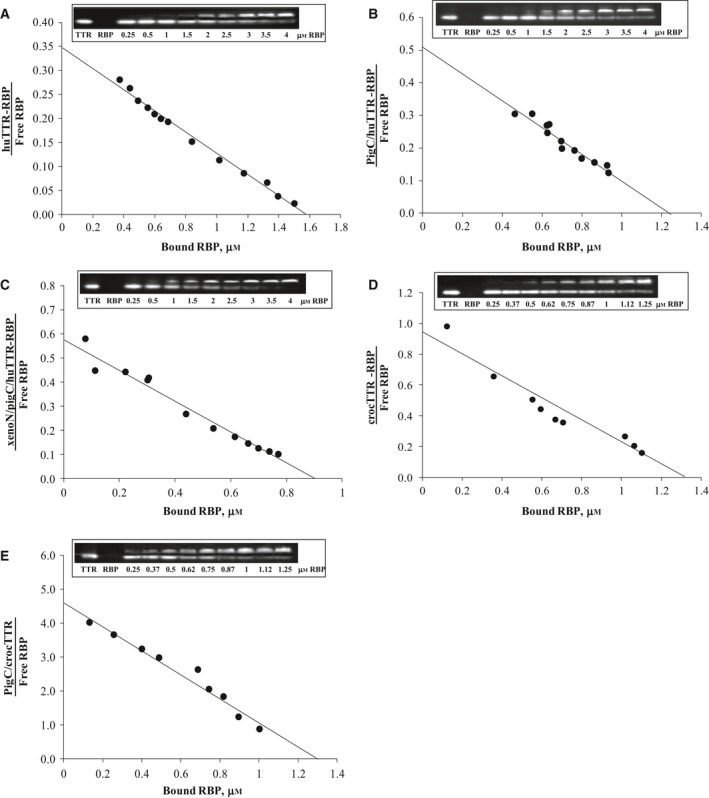
Scatchard plots of the specific binding with human RBP of human TTR (A), pigC/huTTR (B), xenoN/pigC/huTTR (C), *C. porosus* TTR (crocTTR) (D), and pigC/crocTTR (E). Bound and free TTRs were determined by western blot analysis followed by ECL, and the intensities were used to calculate the *K*_d_ of the binding. The inset in each Scatchard plot shows a representative ECL blot of the studied TTR.

To study the effect of longer and more hydrophobic C‐terminal sequence on the binding to human RBP and compare the effect with that of the N‐terminal sequence, purified wild‐type TTRs and chimeric TTRs was incubated with human RBP at various concentrations, at 4 °C for 2 h. Then, the free and bound (in the TTR–RBP complex) forms of TTR were separated by native PAGE. Band intensities of each TTR form were determined by western analysis and then followed by ECL, using specific antibody for TTR. By increasing the amount of RBP, it was shown that the intensity of the free form of TTR gradually decreased while the intensity of the bound form increased (Fig. 2), similarly to that previously reported 19. Each studied TTR showed variation in the range of the specifically bound RBP. The binding interaction with RBP of human TTR was detected from ~ 1.5 to 4 μm RBP, while those of xenoN/pigC/huTTR, pigC/huTTR, *C. porosus* TTR and pigC/crocTTR were detected at lower amounts of RBP, i.e. from 0.25 to 4 μm RBP. According to Scatchard analysis (Fig. 3), the *K*_d_ values of the binding interactions between TTR and RBP could be calculated as shown in Table 1. According to the *K*_d_ values, human TTR bound to RBP with the lowest strength.

**Table 1:** *K*_d_ values for the binding between TTRs and human RBP. Results are presented as the mean ± standard error based on *n* replications

Type of TTR	*K*_d_ (μm)	*n*
Human TTR	4.92 ± 0.68	4
pigC/huTTR	3.20 ± 0.35	4
xenoN/pigC/huTTR	1.53 ± 0.38	3
*Crocodylus porosus* TTR	1.42 ± 0.45	3
pigC/crocTTR	0.31 ± 0.04	3

## Discussion

*Pichia pastoris* is an acceptable host for the synthesis of recombinant proteins, in particular for those which require post‐translational modification for their proper function 24, 25. Based on the physicochemical analysis, the studied chimeric TTRs had electrophoretic migration under native conditions faster than ALB in plasma. In addition, the mass of the tetrameric molecule was four times that of the subunit and had cross‐reactivity with antibody specific to TTR. These findings confirmed that all of these TTRs were successfully synthesized in *P. pastoris* and extracellularly secreted as a tetramer without an inappropriate post‐translational modification, particularly glycosylation, and had an authentic conformation similar to TTR in nature.

The *K*_d_ of the binding sites for RBP of human TTR has been reported with different values depending on the method of determination 26, 27, 28. For example, the *K*_d_ values for the first and the second binding sites of human TTR for RBP determined by electrospray ionization combined with time‐of‐flight mass spectrophotometry were 0.19 ± 1.0 and 35 ± 1.0 μm, respectively 29 and the *K*_d_ of the first binding site determined by fluorescence anisotropy was 0.35 μm 30. In addition, the negative cooperative effect between the two RBP binding sites was supported in TTRs from both mammals and non‐mammals 31. In our previous study, the *K*_d_ of the second binding site of human TTR for human RBP was determined 19. Here, by analysis at low RBP/TTR molar ratio, the obtained *K*_d_ for RBP of human TTR was 6‐fold lower than the *K*_d_ that we previously reported for the second binding site, and it was close to the *K*_d_ of the first binding site for RBP of human TTR determined by other methods 30. However, the *K*_d_ of the binding for human RBP of *C. porosus* TTR was very close to that obtained from the analysis at higher TTR/RBP molar ratio 19. These results indicated a similar affinity of the two binding sites and suggested a less negative cooperative effect between the two binding sites for RBP of the reptile TTR compared with human TTR. In addition, the *K*_d_ values of the two binding sites for RBP of *C. porosus* TTR were smaller than those of human TTR, and this indicated a higher strength of binding between TTR and RBP in the heterologous complex (between *C. porosus* TTR and human RBP) than in the homologous complex (between human TTR and RBP), which confirmed our previous observation 19 and the observation for the binding between bovine TTR and human RBP 32.

According to X‐ray crystallography, both TTR and RBP contributed an equivalent number of amino acid residues in the interaction at the binding interface 9. The interactions that stabilized the protein complex were primarily based on the hydrophobic interactions at the center and charge–charge interactions at the periphery of the binding recognition site 28. In addition, amongst the amino acid residues on the TTR subunit that were involved in the interaction and located at the region of TTR–RBP contact, tyrosine at position 114 participated mainly in the hydrophobic interaction at the center of the recognition site 9, 28. In *C. porosus* TTR, all of the amino acid residues involved in the binding interaction are the same as in human TTR except that the residue at position 114 is a phenylalanine, not a tyrosine. Therefore, increasing the accessibility of RBP to the binding site due to the environment created by longer and more hydrophobic N‐terminal sequence 19 together with increasing the hydrophobic interaction at the center of the binding site which was generated by phenylalanine at position 114 possibly accomplished to higher binding affinity for human RBP with less negative cooperativity of *C. porosus* TTR compared to human TTR.

During the evolution of the TTR gene of mammals from reptile‐like ancestors, the primary structure of TTR at the N‐terminal region changed to be shorter and relatively less hydrophobic, and this affected the binding of TTR to THs and RBP 18, 19, 33. Although it changed less than the N‐terminal sequence, the C‐terminal sequence of TTRs in fishes, amphibians, reptiles and birds is more hydrophobic than in those of mammals; in addition, one to three amino acid residues greater length than in human TTR was observed in the C‐terminal sequence of TTRs from swine, amphibians and lampreys 13, 15, 16, 34. In accordance with the X‐ray crystallography that indicated both N‐terminal and C‐terminal segments of TTR are in close proximity at the entrance to the central binding channel for THs 35, we proposed the evolutionary changes in the C‐terminal sequence of the TTR subunit also affected the accessibility of ligands such as RBP to the central binding channel and the functions of TTR that particularly depended on the interactions of the residues in the C‐terminal region. Herein, two human chimeric TTRs (pigC/huTTR and xenoN/pigC/huTTR) and one *C. porosus* chimeric TTR (pigC/crocTTR) were constructed and the affinities of the binding for human RBP were compared. In comparison with their wild‐type TTRs, pigC/huTTR and pigC/crocTTR, in which only C‐terminal sequences were changed to that of *S. scrofa* TTR, had *K*_d_ values 1.5‐ and 4.6‐fold lower, respectively, indicating an increase of the binding strength to human RBP of these chimeric TTRs. Therefore, a positive effect of a longer and more hydrophobic C‐terminal sequence of TTR on the affinity of its binding with RBP was suggested, because the binding interactions between TTR and RBP in both homologous (pigC/huTTR and human RBP) and heterologous (pigC/crocTTR and human RBP) complexes were increased. The increased hydrophobic environment that is produced by a longer and more hydrophobic C‐terminal sequence of TTR increases charge–charge interactions at the periphery of the TTR–RBP binding interface, and consequently, increases in the stability of the hydrophobic interaction at the center of the binding site could be a mechanism of increasing the strength of the binding of the chimeric TTRs to RBP. In comparison with human TTR and pigC/huTTR, the *K*_d_ values of the binding for RBP of xenoN/pigC/huTTR, in which N‐terminal and C‐terminal sequences were simultaneously changed to longer and more hydrophobic ones, were 3.3‐ and 1.5‐fold lower, respectively. This should indicate a comparable effect of the evolutionary change of the C‐terminal sequence (from longer and more hydrophobic as observed in *S. scrofa* TTR to shorter and less hydrophobic as observed in human TTR) to the change of the N‐terminal sequence (from longer and more hydrophobic as observed in *X. laevis* TTR to shorter and less hydrophobic as observed in human TTR) on the binding of TTR to RBP.

## Author contributions

PP conceived and supervised the study; PP and LL designed the project; RP and SK performed the experiments; RP, SK and PP analysed the data; RP and SK wrote the manuscript; PP and LL revised the manuscript.
